# Clinical Implications of Mismatch Repair Deficiency in Pancreatic Ductal Adenocarcinoma

**DOI:** 10.1002/cam4.70960

**Published:** 2025-05-14

**Authors:** Zachary Kaplan, Elizabeth Prezioso, Aditi Jain, Harish Lavu, Charles J. Yeo, Wilbur B. Bowne, Avinoam Nevler

**Affiliations:** ^1^ Sidney Kimmel Medical College Philadelphia Pennsylvania USA; ^2^ Jefferson Pancreatic, Biliary, and Related Cancer Center Sidney Kimmel Cancer Center Philadelphia Pennsylvania USA

**Keywords:** clinical outcomes, DNA repair, microsatellite instability, mismatch repair, pancreatic cancer, pancreatic ductal adenocarcinoma, TMB

## Abstract

**Background:**

Pancreatic cancer is a highly aggressive and lethal disease, characterized by a limited response to chemotherapy and overall poor prognosis. Pancreatic cancers with a distinct mismatch repair deficiency, although relatively rare, have been shown to be associated with markedly better outcomes in comparison. Furthermore, whereas pancreatic cancers are generally unresponsive to current immunotherapy, this specific group of tumors has been shown to have a notable susceptibility to immune checkpoint inhibitors.

**Aims:**

In this review, we aim to summarize the relevant literature regarding mismatch‐repair associated pancreatic cancers, the impacted biological mechanisms, and the resulting vulnerabilities for potential opportunistic immunotherapeutic treatment approaches. We will also review the current clinical studies assessing survival outcomes of mismatch repair deficient pancreatic cancers and ongoing clinical trials in this emerging field.

**Results and Conclusions:**

Patients with dMMR/MSI‐H pancreatic cancers harbor a distinct phenotype that has increased immune activation, greater responsiveness to immune checkpoint inhibitor therapy and better overall survival when compared to other pancreatic cancers. Although this molecular subtype makes up a small minority of cases, emerging data suggest immunotherapy may offer benefit to these patients.

## 1 Introduction

Patients diagnosed with pancreatic cancer often face a grim prognosis and limited treatment options. Due to the lack of an effective screening test for pancreatic cancer, it is frequently diagnosed at an advanced stage of disease. Furthermore, this cancer displays marked resistance to standard chemotherapy regimens and radiotherapy protocols, leading to a lack of effective treatment options. Surgical resection of early‐stage pancreatic cancer followed by chemotherapy currently is the most effective treatment option for pancreatic cancer [1]. In 2024 it was estimated that 66,440 new cases of pancreatic cancer were diagnosed in the United States along with 51,750 pancreatic cancer‐associated deaths, ranking pancreatic cancer as the third leading cause in cancer mortality [2]. The majority of cases of pancreatic ductal adenocarcinoma (PDAC) have proficient mismatch repair (MMR) tumors, with microsatellite stable status and low tumor mutational burden (TMB) [3]. However, in approximately 1% of cases of PDAC, TMB has been shown to closely correlate with microsatellite instability and defects in MMR. Such deficiencies in MMR are also found in subsets of colorectal, endometrial, ovarian, gastric, and small bowel cancers [4]. Promising advances have now emerged in the treatment of pancreatic cancer in this select group of patients leveraging deficient MMR mutations, utilizing the consequences of altered carcinogenesis pathways to target immunotherapeutic approaches to improve survival.

## 2 What Is Mismatch Repair?

**FIGURE 1:**
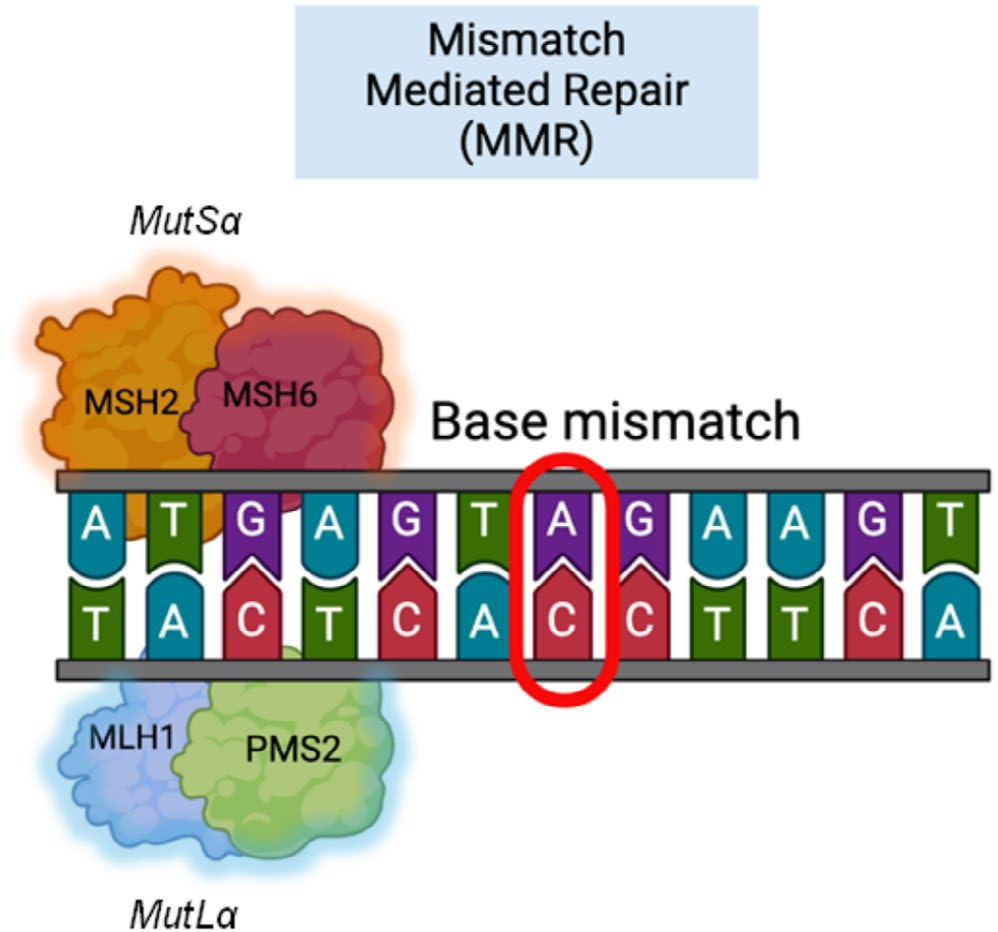
MMR enzymes forming active complexes (MSH2‐MSH6 and MLH1‐PMS2) to identify and repair a base mismatch [9].

MMR is a critical DNA repair pathway that maintains genome stability by correcting base–base mismatches that occur during replication and recombination [5]. The MMR system, first described in bacteria by Su and Modrich [6], consists of a *mutS:mutL* protein system. They found that the *mutS*‐encoded protein binds to single base pair mismatches in DNA. The human homologs of the bacterial MMR system were later discovered by Fishel et al. [7] describing a repair mechanism coordinated by several key proteins, such as *MSH‐2*, *MSH3*, and *MSH6*, which are homologs of *mutS*, and *MLH‐1*, *MLH3*, *PMS1*, and *PMS2*, which are homologs of *mutL*. The mechanism of action was shown to consist of a formation of cascading repair complexes. The *mut*S acts as a sensor that identifies the mismatch, resulting in a conformational change in *mut*S. Once the *mut*S has identified the error, the *mut*L system binds as a facilitator to activate the repair cascade, employing factors, such as *mut*H and others to create single‐stranded nicks, incise, and repair the mismatch. *MSH2‐MSH6* complexes repair single base–base and 1–2 base insertion–deletion (IDL) mismatches. *MSH2‐MSH3* complexes repair some single base IDLs and IDLs ≥ 2 bases. *MLH1‐PSM2* complexes coordinate events from mismatch binding by *mutS* homologs to DNA repair synthesis. The function of the *MLH1‐MLH2* complex in humans is currently unknown. Last, the *MLH1‐MLH3* complex suppresses some IDL mutagenesis and participates in meiosis [8]. The *MSH2‐MSH6* complex acts as *mutSɑ* and the *MLH1‐PMS2* complex acts as *mutLɑ*, as shown in Figure 1. *MUTYH* is another factor. *MUTYH* is a human homolog of the bacterial gene MutY that is involved in DNA repair. It interacts with MMR gene products, but its primary mechanism is base excision repair. The MUTYH gene product is a DNA glycosylase enzyme that is responsible for identifying and repairing mismatches by removing adenines that are mismatched with guanines or 7,8‐dihydro‐8‐oxo‐deoxyguanines [10]. It has been shown to interact with *MSH6* on the *MSH2/MSH6* complex, which enhances the DNA binding and glycosylase activities of *MUTYH*, leading to the repair of these specific mismatches [11].

## 3 Consequences of Mismatch Repair Deficiency

Deficiencies in the MMR system can lead to hypermutability and microsatellite instability. Microsatellites are short, tandemly repeated DNA sequences found throughout the genome. These segments of DNA are typically more prone to mismatch errors during replication due to their repetitive nature and are typically fixed by the MMR system. Therefore, MMR deficiency renders these sequences of DNA specifically susceptible to mutations that can be passed down to the next generation of cells during mitosis [12]. Tumors labeled microsatellite instability high (MSI‐H) contain a large burden of microsatellite instability measured by immunohistochemistry (IHC), polymerase chain reaction (PCR), or next‐generation sequencing (NGS) [12, 13]. The currently gold standard for identifying MSI‐H tumors is PCR; however, visualization and confirmation via IHC are also recommended [14]. Tumors with low levels of microsatellite instability are deemed MSI‐low (MSI‐L), whereas tumors with no microsatellite instability are microsatellite stable (MSS). MSI‐L and MSS tumors typically arise through the chromosomal instability carcinogenesis pathway, wherein oncogenic mutations occur in sequence, eventually resulting in loss of heterozygosity and aneuploidy [15, 16]. Tumor mutational burden (TMB) represents a quantitative measure of the number of somatic mutations per megabase of the genomic sequence. TMB is associated with defects in DNA repair mechanisms including MMR—In a cohort of 385 patients with sporadic PDAC, 20 patients were classified as having a high TMB, 5 of which were considered hypermutated with ≥ 12 somatic mutations/Mb. Of those 5 hypermutated samples, 4 were identified as having dMMR tumors using IHC [17]. High TMB has been shown to effectively predict a beneficial response to immune checkpoint inhibitor therapy [18, 19]. Similarly, MSI‐high status has been shown to positively correlate with TMB, serving as a predictive biomarker for favorable response to immunotherapeutic strategies targeting PD‐1 [20].

A well described clinically significant consequence of MMR deficiency is the increased risk of oncogenic events and development of cancer [21]. Lynch syndrome (LS), also known as hereditary non‐polyposis colorectal cancer (HNPCC), is an autosomal dominant germline mutation in classic MMR genes, such as MSH2, MSH6, MLH1, and PMS2 that is characterized by MSI‐H and higher mutational rates. It accounts for 3%–5% of all colorectal cancers and is the most common hereditary form. It is also associated with an increased risk of developing endometrial (15%–71%), gastric (0.2%–13%), small intestine (0.4%–12%), pancreatic (1.3%–3.7%), ovarian (4%–20%), and upper urinary tract (0.2%–25%) cancers [21, 22, 23]. All patients diagnosed with LS or at risk for LS (first degree relative affected) are advised to receive genetic counseling and undergo routine cancer screenings for early detection. Current United States Multi‐Society Task Force (USMSTF) recommendations advise colonoscopy every 1–2 years beginning at age 20–25 years for colon cancer screening, gynecological examination with transvaginal ultrasound, endometrial sampling, and CA‐125 levels every year from the age of 30–35 for endometrial and ovarian cancer screening, esophagogastroduodenoscopy every 1–3 years from the age of 30–35 years for gastric cancer screening, and urinalysis and urine cytology every year from the age of 30–35 years for urothelial cancer screening [24]. Patients with PDAC associated with LS have higher mutation numbers than patients with somatic mutations in MMR genes which corresponds with longer overall survival in the LS patients [25]. Another example of a high risk population includes individuals who inherit a mutated MMR gene from both parents, in contrast to LS where only one gene is mutated, such that the recipient develops a constitutional mismatch repair deficiency (CMMRD). This is a rare disease with an incidence of 1 per million individuals, which leads to greatly increased risk of multiple malignancies starting from a young age [26, 27]. Based on a study done by Durno et al., the median age of first cancer diagnosis in patients with CMMRD was 9.2 years. Patients with this syndrome are most likely to develop CNS tumors (44%), followed by GI (27%), and hematologic malignancies (19%). Adult tumors, such as colorectal, breast, and genitourinary tumors were observed at a median age of 17.9 years in 31% of patients. Of the patients who survived their initial cancer, 69% developed metachronous cancers [28]. Moreover, MMR mutations are not limited to inherited disorders and can appear sporadically in MMR genes, leading to different types of cancer, including colorectal, lung, gastric, and pancreatic cancer. Likewise, mutations in MUTYH that inactivate methylation of the MLH1 gene can also lead to a similar MMR deficient phenotype [29, 30]. Interestingly, past reports have noted that patients with LS‐associated colorectal cancer present with tumors with greater immune infiltration and activation, along with better overall survival rates than patients with sporadic MMR deficient (dMMR) colorectal cancer. This observation could potentially be attributed to higher somatic mutational burden and immune recognition of increased neoantigen load [31]. Despite this difference in immunogenicity, there is no difference in response rate to immune checkpoint inhibitors between LS‐associated and sporadic MSI cancer patients [32].

## 4 Effect of MMR Status on Immunogenicity

The mutation of MMR proteins, such as MSH2, MLH1, MSH6, and PMS2, resulting in loss of function and shifts in the translational reading frame of microsatellites leading to production of more neoantigens and resulting in increased TMB [33]. In turn, these neoantigens are recognized by the immune system as foreign and can induce an immune response [34]. Germano et al. conducted a seminal study with genetic inactivation of MLH1 in colorectal, breast, and pancreatic mouse cancer cells. They found that tumors deficient in MMR had increased tumor mutational burden and continuous production of diverse neoantigens compared to MMR proficient tumors that displayed low tumor mutational burden and limited neoantigen expression. As a result, dMMR tumors also demonstrated increased immune surveillance compared to the MMR proficient tumors [35]. The mechanisms by which the tumors produce neoantigens include aberrant gene expression, abnormal posttranscriptional modifications, viral infection, and production of mutant peptides [36]. These observations are in accord with recent clinical studies utilizing immune‐checkpoint inhibitors, such as PD‐1 inhibitors, that demonstrated encouraging results as a promising treatment strategy for patients with dMMR malignancies, independent of the tumor subtype [37].

Immune checkpoint inhibitors, typically anti‐CTLA4 and anti‐PD1, act by blocking inhibitory signals that dampen the immune system in response to T cell activation. CTLA4 is an inhibitory receptor expressed on the surface of T cells that competes with the excitatory CD28 receptor [38, 39]. CTLA4 is upregulated upon T cell activation to prevent over‐responsiveness of the immune system [40]. Blocking CTLA4 using immunotherapy increases the native immune response to tumor antigens. PD1 is a protein involved in programmed T cell death that is upregulated on exhausted T cells to suppress the immune response [41, 42, 43, 44, 45]. PD1 receptors are activated by PDL‐1 and PDL‐2, which are ligands expressed on antigen presenting cells (APCs) and cancer cells [44, 45]. PDL‐1 and PDL‐2 are both upregulated in cancerous tissues, which leads to accelerated tumor growth by way of modulating the immune response [46, 47, 48, 49, 50]. Treating cancers with PD‐1/PDL‐1 inhibitors increases the T cell response, which results in decreased tumor growth [46]. There is a significant association between PDL‐1 expression and MLH1/MSH2 loss [51].

Le et al. assessed PD‐1 inhibitors in the treatment of MMR deficient colorectal and non‐colorectal solid cancers [52]. Analysis of their pancreatic cancer sub‐population shows a clear radiographic response in 6 of 8 patients. In 2020, Fraune et al. later conducted a study on 480 samples of PDAC and identified four samples that were deficient in MMR. Remarkably, among collected study samples containing either a loss of MSH6, MSH3, or MSH2, all were found to have a significantly higher density of CD8+ lymphocytes than the samples without an MMR deficiency, suggesting an increased immune response [53]. In 2015, Merck Sharp & Dohme began a clinical trial of pembrolizumab, an immune checkpoint inhibitor, for dMMR/MSI‐H non‐colorectal cancer patients who were unresponsive to prior therapy. This study included 22 patients with dMMR/MSI‐H metastatic pancreatic cancer with an overall response rate of 18.2%, and a median progression‐free survival and overall survival of 2.1 and 4 months, respectively. They noted a median duration of response of 13.4 months [54]. These encouraging results demonstrated that immune checkpoint inhibitor therapy could be used as a potentially effective treatment strategy for dMMR/MSI‐H pancreatic cancer and other dMMR/MSI‐H cancers. Pembrolizumab is indicated for the treatment of advanced pancreatic cancer, as in 2017 it was approved by the FDA for treatment of adults and pediatric patients with unresectable or metastatic, MSI‐H or dMMR solid tumors that have progressed following prior treatment and who have no satisfactory alternative treatment options regardless of tissue of origin [55].

## 5 Testing of Mismatch Repair Deficiency and Microsatellite Instability

Testing for MMR deficiency and microsatellite instability is recommended in cases with clinical suspicion of LS and in cases of LS‐associated cancers [56]. As described in Table 1, there are several testing methods used for the detection of MMR deficiency. The American Society of Clinical Oncology has endorsed the testing guidelines of the College of American Pathologists [61]. Testing can include immunohistochemical (IHC) analysis, MSI detection by PCR, and MSI detection by next generation sequencing (NGS). The testing recommendations for MSI in colorectal cancer are outlined in Table 2. In colon cancer, gastroesophageal cancer, and small bowel cancer, the current recommendations support the use of MMR‐IHC and MSI detection through PCR rather than the use of MSI‐NGS for the detection of DNA MMR deficiency due to cost‐effectiveness and diagnostic effectiveness [63, 64]. In contrast, based on the most recent NCCN guidelines for pancreatic adenocarcinoma, testing should be considered for potentially actionable somatic findings such as MSI or MMR deficiency using NGS due to efficiency with limited tissue samples, detection of a wide range of genetic alterations, and the low prevalence of dMMR in pancreatic cancer [65, 66, 67]. Immunohistochemical (IHC) staining of tumor tissue for protein expression targets the 4 MMR genes frequently mutated in LS: MLH1, MSH2, MSH6, and PMS2. There is a 5%–10% false‐negative rate with IHC testing, and abnormal results are recommended by the NCCN to be followed with germline or tumor DNA testing. Abnormal MLH1 IHC results should also be followed by testing the tumor tissue for MLH1 methylation. MSI detection with PCR amplification utilizes panels testing several mono/dinucleotide or pentanucleotide genetic loci. The specificity and sensitivity of the detection of LS by PCR‐based methods for MSI are 90% and 85%, respectively. There is a 5%–15% false‐negative rate with MSI testing. MSI detection by NGS, though possible for colorectal cancers, is not recommended for use in other cancers. NCCN guidelines note that NGS assessment of MSI is possible if the laboratory has validated the assay for use in the cancer in which it is being used [65].

**TABLE 1:** Methods of detecting MMR deficiency.

Method	Summary of technique
Immunohistochemistry (IHC)	Involves the detection of antigens on formalin‐fixed, paraffin‐embedded tissue sections using antibodies of choice. It is largely accepted as the most basic method for protein expression detection [57]
Polymerase chain reaction (PCR)	A laboratory nucleic acid amplification technique that identifies the presence of a pre‐isolated gene sequence in a tissue sample. Useful in detecting gene mutations due to its high sensitivity [58]
Next‐generation sequencing (NGS)	A large‐scale RNA and DNA sequencing technique characterized by a “massively parallel” data generation that reads billions of short segments of genetic material simultaneously. Useful in characterizing tumor mutational burden [59, 60]

**TABLE 2:** Testing guidelines for patients with suspected hereditary non‐polyposis colorectal cancer (Lynch syndrome) [62].

Revised Bethesda criteria for testing colorectal cancer for MSI
1. Colorectal cancer diagnosed in a patient less than 50 years of age
2. Presence of synchronous, metachronous colorectal, or other HNPCC‐related tumors
3. Colorectal cancer with MSI‐H histology diagnosed in a patient who is less than 60 years of age
4. Colorectal cancer diagnosed in one or more first‐degree relatives with an HNPCC‐related tumor, with one of the cancers being diagnosed under age 50 years
5. Colorectal cancer diagnosed in two or more first‐ or second‐degree relatives with HNPCC‐related tumors, regardless of age

## 6 MMR Deficiency in Pancreatic Cancer

Studies throughout the years have reported the prevalence of dMMR/MSI‐H PDAC to range between 0% and 10%, with a few reporting rates as high as 22%. These numbers vary greatly based on geographic location, sample study sizes, and the evolving testing methods [68, 69]. Recent studies have now demonstrated that patients with MMR‐deficient pancreatic cancer have earlier onset tumors, distinct histological patterns, increased immunogenicity, improved response to immunotherapies, and longer overall survival when compared to MMR‐proficient pancreatic cancer (as shown in Table 3). Grant et al. conducted a retrospective cohort study on patients with pancreatic cancer. They found that 12 out of 1213 (0.98%) patients in the cohort had dMMR tumors confirmed by immunohistochemistry (IHC) or whole‐genome sequencing (WGS); these patients had longer overall survival after surgery [73]. Karamitopoulou et al. likewise analyzed tumor mutational burden in 166 cases of PDAC and found 12 high TMB cases (7.2%). These tumors similarly had higher densities of T cells and greater immune pathway activation than all other cases. Among this study group, all MSI‐H tumors were found to be TMB‐high [82]. Nasar et al. conducted a retrospective study on patients who underwent surgical resection of PDAC between 1990 and 2023. They used genetic sequencing data from 963 patients to identify 17 cases of dMMR/MSI‐H or LS‐associated surgically resected PDAC. Remarkably, the median survival of these patients was 12 years as compared to only 1.9 years in the control group with 5‐year survivals of 81% and 18%, respectively. This finding suggests that dMMR/MSI‐H status is a positive predictive biomarker [70]. Moreover, Quintanilha et al. retrospectively evaluated 21,932 patients with PDAC, 293 (1.3%) of whom had a high TMB [85]. High TMB was associated with increased prevalence of mutations in the homologous DNA repair pathway and the MMR pathway, along with decreased rates of KRAS mutations. A total of 51 patients received immune checkpoint inhibitor (ICI) therapy, 41 patients with low TMB and 10 patients with high TMB. Of the patients who received ICI therapy, those with high TMB had an improved median overall survival (OS) (25.7 vs. 5.2 months) with a time to respective treatment discontinuation (TTD) of 12.4 vs. 2.3 months. Notably, for patients treated with the standard chemotherapy regimen, there was no difference in OS or TTD between the high TMB and low TMB groups. Sixty percent of the high TMB patients had longer OS when treated with ICI therapy compared to the standard chemotherapy regimen (25.7 vs. 6.6 months) [85]. In a recent study by Taïeb et al., the investigators found similar responses to ICI therapy in dMMR/MSI‐H advanced PDAC. They identified 31 cases of dMMR/MSI‐H advanced PDAC who received either single‐agent anti‐PD1 antibodies, a combination of nivolumab and ipilimumab, or the combination of immunotherapy plus chemotherapy. They found that 15 (48.4%) patients had an objective response and 6 (19.4%) patients had stable disease resulting in a disease control rate of 67.7%. The median progression‐free survival (PFS) was 26.7 months and there were no grade 3 or 4 adverse effects observed [87]. At last, Coston et al. retrospectively explored the efficacy of cytotoxic chemotherapy versus ICI therapy in 32 patients with dMMR/MSI‐H pancreatic cancer from 2009 to 2023 at the Mayo Clinic. They reported an overall response rate (ORR) of 75% (20% complete response) to ICI therapy as compared to 30% ORR to cytotoxic chemotherapy [88].

**TABLE 3:** Literature review assessing the link between mismatch repair status and oncologic outcomes in pancreatic ductal adenocarcinoma.

Study	Results and conclusions
Nasar et al., 2024 [70]	The median survival time of patients with MSI‐H, dMMR, or LS‐associated PDAC was 12 years vs. 1.9 years in patients with MSI‐stable, MMR proficient, and non‐LS‐associated PDAC. Five‐year survival was also 81% vs. 18%, respectively
Zalevskaja et al., 2023 [71]	Patients with LS‐associated pancreatic cancer have better overall survival than sporadic pancreatic cancer
Nakata et al., 2002 [72]	Postresection MSI‐positive patients have significantly longer survival and higher leukocyte invasion than MSI‐negative patients
Grant et al., 2021 [73]	MMR‐deficient pancreatic cancer patients had longer postsurgical survival, higher TMB, and greater neoantigen loads. MMR‐deficient tumors were also less likely to have traditional PDAC mutations such as KRAS and SMAD4, and more likely to have mutations in ACV2RA and JAK1 which are associated with MSI
Prezioso et al., 2024 [74]	dMMR status in PDAC is associated with significantly improved overall survival after resection
Yamamoto et al., 2001 [75]	Patients with MSI‐high pancreatic tumors have a longer overall survival time than MSI‐low and microsatellite stable pancreatic tumors. MSI‐high tumors also have poor differentiation and wild‐type KRAS and p53
Dong et al., 2011 [76]	MMR gene variants are significantly associated with increased overall survival in resectable, local advanced, and metastatic and pancreatic cancer
Cloyd et al., 2017 [77]	Patients with localized dMMR PDAC that underwent surgical resection had a 100% 5‐year overall survival rate compared to 25% in all other cases of localized PDAC. Patients with metastatic dMMR PDAC showed a similar improved outcome with a median overall survival of 16.5 months compared to 11.1 in other patients with PDAC
Dong et al., 2009 [78]	Single nucleotide polymorphisms (SNPs) of MMR genes can have statistically significant effects on PDAC tumor response to preoperative therapy, tumor resectability, and overall and disease‐free survival
Liang et al., 2018 [79]	In patients with PDAC who underwent surgery alone, patients with dMMR tumors have better overall survival than patients with pMMR tumors
Krykylva et al., 2022 [80]	Early‐onset pancreatic cancer with MMR deficiency is highly suggestive of a germline mutation which can be characterized as LS
Luchini et al., 2021 [66]	MMR‐deficient PDAC is associated with medullary and mucinous/colloid histology and wild‐type KRAS and p53. These tumors more commonly have JAK mutations that are associated with MSI
Goggins et al., 1998 [81]	MMR‐deficient PDAC tumors show a distinct histological pattern: poorly differentiated, expanding borders, and syncytial growth pattern. These tumors also have wild‐type KRAS
Karamitopoulou et al., 2022 [82]	Pancreatic tumors with high TMB demonstrate greater T cell density when compared to all other cases. The cytotoxic T cells are found closer to the tumor cells and T helper cells are closer to dendritic cells. High‐TMB tumors also show increased immune pathway activation when compared to TMB‐low tumors. Patients with high TMB pancreatic cancer had prolonged survival compared to all other cases
Luchini et al., 2019 [83]	MMR deficiency and MSI are predictive biomarkers for responsiveness to immunotherapy
Lawlor et al., 2021 [3]	MMR‐deficient cases of PDAC are associated with mucinous/colloid and medullary histology, and show promising responses to immunotherapy
Chakrabarti et al., 2022 [84]	Use of liquid biopsy for detection of MSI‐high pancreatic cancer has prognostic value and improved outcomes
Quintanilha et al., 2023 [85]	Patients with high TMB pancreatic cancer had greater median overall survival when treated with immune checkpoint inhibitors when compared to patients with low TMB pancreatic cancer
Mucileanu et al., 2021 [86]	MMR deficient pancreatic cancer is associated with high levels of neoantigens which could lead to a favorable response to anti‐PD1/PDL1 therapy
Taïeb et al., 2023 [87]	Immune checkpoint inhibitors such as nivolumab (PD‐1 inhibitor) and ipilimumab (CTLA‐4 inhibitor) are effective (26.7 months median progression‐free survival) and well‐tolerated in patients with advanced MMR‐deficient PDAC
Coston et al., 2023 [88]	Patients with dMMR/MSI‐H pancreatic cancer that underwent surgical resection have lower rates of recurrence when compared to patients with MMR‐proficient pancreatic cancer. Patients with dMMR/MSI‐H pancreatic cancer also showed excellent responses to various immune checkpoint inhibitor therapy regimens with an overall response rate of 75% (20% complete response), which was more effective than cytotoxic chemotherapy with a 30% overall response rate
Marabelle et al., 2020 [54]	Patients with dMMR/MSI‐H pancreatic cancer treated with pembrolizumab (anti‐PD1 antibody) had an objective response rate of 18.2%, a median progression‐free survival of 2.1 months, and a median overall survival of 4 months
Hu et al., 2018 [68]	4 of 7 (57%) patients with dMMR pancreatic cancer had a beneficial response to immune checkpoint inhibitors (1 complete response, 2 partial responses, 1 stable disease)
Noor et al., 2021 [89]	Out of 6 evaluable patients with dMMR PDAC, 3 had an objective response (1 complete and 2 partial) and 2 had stable disease after receiving treatment with either pembrolizumab or ipilimumab/nivolumab combination therapy

However, other studies have highlighted the varying efficacy of immunotherapy in MSI‐H pancreatic cancers. The KEYNOTE‐158 study, which trialed pembrolizumab in patients with MSI‐H non‐colorectal solid tumors, showed an overall response rate of 18.2% in the PDAC cohort [90]. In this study, PDAC exhibited the lowest ORR to pembrolizumab compared to other cancer types, including endometrial, cholangiocarcinoma, gastric, and small intestine. One hypothesis for this varied response is the immunosuppressive nature of the PDAC tumor microenvironment (TME), which is a result of interactions between epithelial tumor cells and surrounding stroma [91, 92]. Current literature shows that several factors, including obesity, modulate the activity of tumor‐associated macrophages (TAMs) in the PDAC TME. TAMs in turn secrete immunosuppressive cytokines such as TGF‐B and IL‐10, among others, that hinder recruitment and activity of effector T cells, regulatory T cells, and cytotoxic CD8+ T‐cells [93, 94]. Various other immune cell subtypes in the PDAC TME, namely myeloid‐derived suppressor cells, Th17 cells, and neutrophils, also contribute to its immunosuppressive nature [95, 96, 97, 98, 99, 100, 101, 102, 103, 104]. Additionally, signaling protein VEGF plays a role in the TME, similarly impairing the recruitment and effector mechanisms of immune cells by interfering with angiogenesis [105]. It is hypothesized that due to these aforementioned factors, the TME renders certain PDAC subtypes largely resistant to immunotherapy. Early evidence suggests that combinatorial therapies, such as ICIs and colony‐stimulating factor‐1 receptor (CSF1R) inhibitors, may combat this resistance [106].

## 7 Future Directions

In 2017, pembrolizumab became the first immune checkpoint inhibitor approved by the FDA for treatment of all dMMR and MSI‐H solid tumors, including pancreatic cancer [55]. Since then, multiple clinical studies have investigated the use of immune checkpoint inhibitors and immunotherapies alone or in combination with chemotherapy to treat MSI‐H or dMMR pancreatic cancers.

NCT02791334 was a phase 1a/1b multi‐center, multi‐arm clinical trial, sponsored by Eli Lilly and company. IT assessed the PD‐L1 antibody, LY3300054 alone and in combination with the TIM3 antibody, LY3321367, in PD‐1/PD‐L1‐naive, MSI‐H/MMR‐deficient advanced solid tumors. Monotherapy with LY3300054 resulted in an ORR of 32.5% and a DCR of 60.0%, whereas the combination treatment yielded an ORR of 45.0% and a DCR of 70.0%. However, only 2 patients out of the entire cohort had pancreatic cancer, greatly limiting the interpretability of the results in the context of PDAC [107].

NCT05093231 is a phase II randomized controlled trial (RCT) on pembrolizumab/olaparib combination therapy for the treatment of metastatic high‐TMB (including dMMR/MSI‐H) pancreatic adenocarcinoma. They are enrolling patients with stage 4 PDAC who have undergone no more than one prior systemic treatment regimen, with objective response rate as the primary endpoint [108]. A group of investigators from Unicancer is conducting a phase II RCT on the use of dostarlimab, an immune checkpoint inhibitor, on locally advanced metastatic dMMR/MSI‐H non‐colorectal/non‐endometrial cancers, including participants that have dMMR/MSI‐H pancreatic cancer. The study is comparing progression‐free survival in patients treated with dostarlimab versus the standard of care chemotherapy regimens [109].

NCT05078866 is a phase 1b/2 multi‐center clinical study, assessing cancer prophylaxis in LS patients with the Nous‐209 vaccine and aimed at improving neoantigen immunogenicity. Similarly, the vaccine consists of multiple neoantigens common to LS‐associated cancers designed to enhance immune surveillance and tumor immunity. The vaccine is being tested on patients with confirmed diagnoses of LS or Lynch‐like syndrome (mutation negative) with no prior history of cancer or systemic treatment of cancer in the past 6 months. The study is expected to conclude in 2025 [110].

NCT05419011 is a multi‐center phase 2 study assessing a combination of N‐803 and Tri‐Ad5 vaccines for the prevention of cancer in patients with LS. N‐803 is an IL15‐IL15Rɑ super‐agonist that can enhance natural killer cells and cytotoxic T cells. This vaccine is designed to increase the immune response to other vaccines including Tri‐Ad5. Tri‐Ad5 is a vaccine regimen that consists of 3 doses that each contain different tumor‐associated antigens found in precancerous and cancerous cells. The endpoints include measurement of the cumulative incidence rate of the composite endpoint of adenomas, advanced adenomas, and colorectal cancer. The study is planned to conclude in 2027 [111].

## 8 Conclusions

Understanding the role of MMR deficiency in tumor mutational burden and microsatellite instability has opened new therapeutic possibilities in the treatment of pancreatic cancer. Patients with dMMR/MSI‐H pancreatic cancer harbor a distinct subtype of pancreatic cancer that has better overall survival, a distinct histopathological appearance, increased immune activation, and greater responsiveness to immune checkpoint inhibitor therapy when compared to other pancreatic cancers. Although this molecular subtype makes up a small minority of cases, emerging data suggest immunotherapy may offer benefit to these patients. There are several open clinical trials exploring the efficacy of immunotherapy for dMMR/MSI‐H pancreatic cancer and vaccines that prevent cancer occurrence in patients with LS. There is potential for further exploration of the development of a targeted therapy that induces dMMR by knockdown of MMR gene expression to combine with immune checkpoint inhibitors to increase survival of all patients with pancreatic cancer.

## Author Contributions

Conceptualization, A.N., and A.J.; methodology, A.N., Z.K., E.P.; investigation, Z.K., and E.P.; writing – original draft preparation, Z.K., E.P., and A.N.; writing – review and editing, A.N., A.J., H.L., W.B.B., C.J.Y.; supervision, A.N. All authors have read and agreed to the published version of the manuscript.

## Conflicts of Interest

The authors declare no conflicts of interest.

## Data Availability

Data sharing not applicable to this article as no datasets were generated or analysed during the current study.
